# An ERP study on the late stage of Chinese metaphor processing

**DOI:** 10.3389/fnhum.2024.1269153

**Published:** 2024-06-07

**Authors:** Xinyi Xu, Jingting Zhang, Yuling Wang, Minghu Jiang

**Affiliations:** ^1^Center for Psychology and Cognitive Science, Tsinghua University, Beijing, China; ^2^College of National Culture and Cognitive Science, Guizhou Minzu University, Guiyang, China; ^3^Artificial Intelligence and Human Language Lab, Beijing Foreign Studies University, Beijing, China

**Keywords:** conventional metaphor, familiarized metaphor, prime-probe, ERP, N400

## Abstract

Psycholinguistic models of metaphor processing remain a subject of debate. A prime-probe design using Chinese materials with a specific time span (300 ms) was applied to test the mechanisms of metaphor processing. Conventional and familiarized metaphors were designed as primes, followed by a probe word semantically related to the prime metaphor (MT), a probe word related to the literal meaning of the final word of the prime metaphor (LT), control/unrelated probe word (UT), or non-word. Event-related potentials (ERPs) elicited by the probes were recorded to examine metaphor processing. In N400, results revealed that UT and LT elicited significantly more negative waveforms than MT in both primes. MTs and LTs showed no difference between conventional and familiarized metaphors, suggesting that metaphorical meaning may be accessed directly, regardless of whether conventional or familiarized metaphors. The results were generally compatible with the direct processing model.

## 1 Introduction

Metaphors, one of the most widely used non-literal expressions and a reflection of our thought and conceptual system, have attracted great attention in recent years. For example, in Chinese, the metaphor “欲望是黑洞 (Desire is a black hole)” employs the term “black hole” not as an astronomical concept existing in outer space but refers to the shared features of desire and black hole, meaning endless and awful. Understanding this metaphor links the topic (desire) and vehicle (black hole), establishes relations between the two semantically remote concepts, and conveys some information different from the literal meaning of the vehicle. As metaphors present a non-literal meaning, interest in exploring the difference between metaphor and literal expressions has caught great attention in the fields of psychology, philosophy, linguistics, and neuroscience. The basic and most widely tested metaphorical structure is “X is Y,” as many complex expressions stem from it (Lakoff and Johnson, 1980). Many discoveries have emerged in studies on metaphor and literal expressions. Recently, comparisons between conventional and novel metaphors have also generated a lot of research, but no consensus has yet been reached.

In psycholinguistics, there are mainly two contrasting views on metaphor processing. One is the three-stage processing model/indirect processing model, put forward by Searle (1979) and Grice (1975), who proposed that before metaphorical meaning is processed, the literal meaning is accessed first and then rejected as inconsistent with the context. By contrast, the direct processing model holds that metaphorical meaning can be accessed directly without the literal meaning being first processed and rejected (Gibbs, 1984; Glucksberg and Keysar, 1990), since there is a dual representation of the vehicle. Namely, the vehicle represents both the domain-specific/literal meaning and the domain-general/higher concept shared by the topic and vehicle. People could automatically and categorically access metaphorical meaning.

Initially, empirical studies supporting the two theories came mostly from measuring the reading time of metaphor and literal sentences. For example, Janus and Bever (1985) found longer reaction times in reading metaphorical than literal expressions, thus supporting the indirect processing model. Similarly, in Cacciari and Tabossi (1988) study, subjects responded faster to the literal meaning related target word than the metaphorical meaning related target word after hearing idiomatic expressions, suggesting that even in conventionalized expressions, the literal meaning is also accessed first. In comparison, other studies found no difference in reaction time between metaphorical and literal sentences (Glucksberg et al., 1982; Gerring and Healy, 1983; Blasko and Connine, 1993) and supported the direct processing model. For example, in one Stroop-like paradigm, participants were asked to judge whether the literal meaning of a sentence made sense. Results showed that the reaction time (RT) of a metaphor's literal and metaphorical meanings showed no difference, suggesting literal and metaphorical meanings can be accessed simultaneously (Gildea and Glucksberg, 1983). Similarly, Blasko and Connine (1993) found that for metaphor rated apt (metaphorical and interpretable), metaphorical meaning could be accessed as fast as the literal meaning, which exists in both familiar and novel metaphors.

However, some researchers claimed that the findings that figurative and literal meanings are accessed simultaneously are largely based on conventional metaphors (Bowdle and Gentner, 2005; Arzouan et al., 2007). With a deeper and closer look at metaphors, some studies reported differences in processing conventional/familiar and novel metaphors. Then, searching for a theory that could explain conventional and novel metaphor processing became a goal.

One noteworthy model is the graded salience hypothesis/GSH (Giora, 1997), which proposed that salient meaning is always accessed first. Salient meaning refers to the first meaning that comes to mind and is characterized by conventionality, familiarity, and frequency, enhanced by prior context. Giora considered metaphorical and literal expression processing to be affected by saliency and claimed that it is not metaphoricity, but saliency determines the processing. In this model, for the conventional metaphor “家庭是港湾 (Family is a harbor),” the listener could directly access the salient metaphorical meaning of “feeling protected,” whereas for the novel metaphor “创新是铁锹 (Innovation is a shovel),” the salient literal meaning will be accessed first. Thus, under this framework, understanding conventional metaphors is expected to take less time than understanding novel metaphors. For example, Klepousniotou and Baum (2005) experimented on ambiguous word comprehension with the prime ambiguous word followed by different targets. They found that only targets related to the dominant meaning were facilitated in the metaphor condition, and the result supported GSH.

Another theory illuminating the processing of conventional and novel metaphors is the career of metaphor (Bowdle and Gentner, 2005), which advocates the concept of mapping (Lakoff and Johnson, 1980) and originated from structure-mapping theory (Gentner, 1983). The central claim is that as metaphors are conventionalized, there is a shift in the mode of processing from comparison to categorization. Specifically, novel metaphors are initially processed comparatively. The literal meaning is accessed first, then rejected, and the metaphorical meaning is accessed. As conventionalization deepens, novel metaphors could turn into conventional, even dead metaphors. In general, conventional figuratives should be comprehended more rapidly than novel figuratives. Bowdle and Gentner (2005) conducted three experiments to illustrate their theory. Experiments from Lai and Curran (2013) also showed that the priming sentences in comparison form could better facilitate the process of novel metaphors, whereas priming sentences in categorization form facilitated conventional metaphors better, giving some evidence to the career of metaphor.

As mentioned above, empirical evidence for these models mostly originated from reading time. These researches raised different results and supported different theories. Even if studies showed similar results, it does not necessarily mean equal effort was paid (Coulson and Van Petten, 2002). Thus, recording reading time/reaction time might not be sufficient to detect differences among different conditions as the potential mechanisms are covered. Event-related potentials (ERPs), a highly time resolution measurement, have been widely applied to metaphor research. The specific components in critical points could inform us more about the underlying cognitive process and reveal the online process. The most tested component is N400, a negative-going wave, peaking at ~400 ms, firstly found in semantic violation in sentences (Kutas and Hillyard, 1980), has recently been reported in metaphor processing (Pynte et al., 1996; Coulson and Van Petten, 2002; Tartter et al., 2002; Iakimova et al., 2005; Arzouan et al., 2007; Lai et al., 2009; De Grauwe et al., 2010; Goldstein et al., 2012; Lai and Curran, 2013). Another worth noted component is P600/LPC, a positive-going wave peaking at ~600 ms, closely related to syntactic processing, elaboration, and integration processes (Neville et al., 1986), which is a reflection of reanalysis in most metaphor studies (Pynte et al., 1996; Arzouan et al., 2007; Goldstein et al., 2012).

However, studies using ERPs to explore the mechanisms of metaphor processing have also failed to reach consistent conclusions. For example, Arzouan et al. (2007) examined novel metaphor processing in adjective word pairs using conventional word pairs (lucid mind), novel word pairs (ripe dream), control word pairs (burning fire), and unrelated word pairs (indirect blanket) in Hebrew and operated a semantic judgment task. Results showed a sequentially increasing N400 in the control word pairs, conventional word pairs, novel word pairs, and unrelated word pairs. They suggested that novel metaphors are more difficult to process than conventional metaphors, and the result supported the indirect processing model in novel metaphor comprehension. A further study on novel word pairs (Goldstein et al., 2012) replicated the same result in N400 in the four conditions and also supported the indirect model in novel metaphor processing. However, Tartter et al. (2002) also examined novel metaphor processing but raised different results. They examined sentence-final words with literal, metaphorical, and anomalous conditions and found no difference between metaphorical and anomalous sentences in the early time window. Although waveforms for metaphorical and literal conditions converged in 300–500 ms, only anomalous sentences elicited a significantly larger N400. It was concluded that the results provided evidence for the direct processing model since the waveforms for novel metaphor and literal expression converged in the N400 time window. Iakimova et al. (2005) conducted experiments on schizophrenia patients and non-patients using the same categories. They also found incongruous endings evoked the most negative N400 amplitudes, whereas metaphors were not more difficult to process than the literals in non-patients. The inconsistency between these four studies may result from material structure and task, as Arzouan et al. (2007) and Goldstein et al. (2012) examined word pairs and operated a semantic judgment task, whereas Tartter et al. (2002) and Iakimova et al. (2005) examined sentences without semantic judgment, which may cause different process mechanisms and lead to opposite conclusions, leaving the process mechanism of novel metaphor unknown and the comparison between conventional and novel metaphors obscure.

In contrast to the above studies, some studies used a prime-target paradigm. Compared with the sentence-final paradigm, this paradigm provided a new way to examine conventional and novel expressions. Through primes, participants pre-fabricated the meaning. The extent to which the meaning has been structured and accessed could be examined by analyzing Target's RT and ERP. For example, in Laurent et al. (2006), participants engaged in reading and lexical decision task to (strong/weak) salient idioms and (figurative/literal) targets, N400 of targets related to the salient meaning of strong salient idioms was less negative than the three other conditions, suggesting salient meanings are accessed automatically, which supported GSH. Contrary to this, Forgács et al. (2014) examined literal conventional, literal novel, conventional metaphor, and novel metaphor in divided visual field experiments. They found novel metaphors were processed just as fast as novel literal expressions, which supported the direct model and was against GSH since if the comprehension of novel expressions is a serial processing of salience, there should be a difference between the two conditions. The same result was also revealed by Forgács et al. (2012). However, our concern regarding Forgács et al. (2012, 2014) was that their conclusions were based solely on behavioral data.

Also using the prime-target paradigm, but Lai and Curran (2013) supported the career of metaphor. Two experiments were conducted to examine conventional and novel metaphor processing. Participants read literal expressions, conventional metaphors, novel metaphors, and anomalous sentences preceded by primes with related or unrelated expressions. In Experiment 1, related sentence primes reduced the N400 for conventional metaphors, whereas novel metaphors showed no difference with related and unrelated primes. In Experiment 2, related primes were in the form of comparisons. The two kinds of metaphors both elicited less negative N400 in the related condition than in the unrelated condition. Primes in comparison form better facilitated novel metaphor processing, whereas the categorization prime was more effective in priming conventional metaphors. This difference in prime effectiveness suggested that people understand novel metaphors using comparison and comprehend conventional metaphors using categorization, which supported the career of metaphor.

In summary, ERP studies on metaphors varied in paradigm, task, materials, and results. Most studies focused on examining the end word of a sentence since the end word entails sentence type and initiates metaphor processing directly. By contrast, the results of these studies were controversial. Within the category of novel metaphor, researchers selected different kinds of materials (e.g., word pairs and sentences). Even though they adopted sentences as materials, sentence structures were different among various studies. Some used basic, and others used complex structures, leading to inconsistent results. It is hard to suggest what processing mechanism underlies novel metaphors. Even less could be said about the comparison between conventional and novel metaphors. In addition, tasks for participants were different; some studies asked participants to read presented sentences without any judgment (Pynte et al., 1996; Tartter et al., 2002; Yang et al., 2013), some asked participants to judge whether the presented materials made sense (Arzouan et al., 2007; Lai et al., 2009; Lai and Curran, 2013) and some asked participants to make lexical decision task (Klepousniotou and Baum, 2005). Different tasks may lead to different results, and these studies have not received consensus yet. In addition, the control factors were not identical. For example, some studies lacked the scale for familiarity (Pynte et al., 1996), some neglected the frequency of the target (Sun et al., 2022), and some overlooked aptness (Lai et al., 2009; Lai and Curran, 2013). In addition, some studies presented the same novel metaphor primes two or more times (e.g., Sotillo et al., 2004; Cardillo et al., 2012; Sun et al., 2022), novel metaphors may have lost their novelty through repetition in the process and become familiarized/conventionalized metaphors (still distinct from the conventional metaphors). Thus, strictly speaking, novel metaphors in these studies should not be called novel metaphors but rather familiarized/conventionalized metaphors (between conventional and novel metaphors). To the best of our knowledge, a comparison between conventional metaphors and familiarized metaphors has not been done before.

Considering these deficiencies, we planned to conduct an ERP study to examine whether there was a difference between conventional and familiarized metaphors in a prime-probe design with the basic structure “X is (a) Y” and more specific control factors. Compared with the sentence-final paradigm, the prime-probe design could set a specific time span to examine the meaning process. At present, studies using prime-target/probe with ERPs in metaphor are limited (Sotillo et al., 2004; Sun et al., 2022). We will use the prime-target/probe paradigm with a lexical decision task (Rubio Fernandez, 2007; Klepousniotou et al., 2012; Al-Azary and Katz, 2021) to explore the exact processing phase and set a specific time span (300 ms) between the prime and target/probe word to specifically locate at the late stage of metaphor processing, just as Cacciari and Tabossi (1988) and Blasko and Connine (1993) did. Contrary to the two behavioral studies, we used ERPs to examine metaphor processing instead of only recording RT. This study is the first ERP study detecting metaphor sentence processing with three kinds of probe words (literal related, metaphorically related, and unrelated word) primed by conventional and familiarized metaphors. This study aims to explore to what extent the meaning of Chinese metaphors has been processed at a later stage (the time from vehicle presentation to probe presentation lasts for 500 ms/ SOA = 500 ms), whether the metaphorical meaning has been accessed and whether understanding conventional and familiarized metaphors induce different mechanisms. Conventional and familiarized metaphors are designed as prime, words semantically related with the metaphorical meaning of metaphor (MT), semantically with the literal meaning of vehicle (LT), and unrelated words (UT) are designed as the probes to test if literal meaning is accessed and then rejected before metaphorical meaning is processed and whether the processing mechanisms of familiarized and conventional metaphors were different. That is, we examine the processing for metaphors by presenting probe words following them instead of examining the metaphors themselves. We mainly focused on the N400 component of the probe words (Sun et al., 2022) and briefly looked at P600.

Our hypothesis is as follows: if the indirect processing model holds, LT and UT are supposed to elicit different waveforms in N400 under both conventional and familiarized metaphor priming conditions, as participants are supposed to process the literal meaning of the prime metaphor before the target is presented. A priming effect will be executed, and we will observe a more negative N400 on UT compared to LT. UT is also supposed to elicit more negative N400 than MT, as metaphorical meaning is supposed to be accessed at a later stage of sentence comprehension, while UT is unrelated to either the literal or metaphorical meaning of the metaphor prime. If the direct model holds, literal meaning does not need to be accessed first, metaphorical meaning could be accessed directly. Thus, LT and UT will elicit more negative N400 than MT. If GSH holds, the metaphorical meaning of the conventional metaphor will be accessed first, MT will trigger a less negative N400 than UT under the conventional metaphor condition. On the other hand, the literal meaning of the familiarized metaphor will be accessed first so that LT will trigger a less negative N400 than UT under the familiarized metaphor condition. As for the scalp activation, we are supposed to observe centroparietal negativity in N400 and central positivity in P600, and the largest amplitude may be located on midline electrodes.

## 2 Method

### 2.1 Participants

A total of 28 native Chinese students (mean age = 22.8 years, range = 18–25 years; 14 males) from Tsinghua University participated in this study. All participants were right-handed with normal or corrected-to-normal vision before the experiment. Handedness was assessed by the Edinburgh Handedness Survey (Oldfield, 1971). None of them reported a history of neurological or psychiatric impairments. Informed consent was obtained from all the participants according to the Declaration of Helsinki. Data from three participants were excluded because of low correct ratios (*n* = 1, the correct ratio was 0.77) and noisy EEG data (*n* = 2, the ratio of noisy trials was 0.56 and 0.67), leading to a final number of 25 participants (12 males, 13 females).

### 2.2 Stimuli

For the formal experiment, 720 prime-probe pairs of Chinese phrases were designed. These stimuli consisted of three prime categories–conventional metaphors, familiarized metaphors, and literal expressions–with 240 pairs in each group. For conventional metaphors, 40 metaphors were paired with MT (metaphorical meaning), LT (literal meaning), and UT (unrelated) separately, forming 120 prime-probe pairs, and the remaining 120 metaphors were paired with non-words as fillers (Blasko and Connine, 1993). The same design was also applied to familiarized metaphors. For literal expressions, all primes have literal meanings, and half were paired with words, and half were paired with non-words.

Specifically, as shown in Table 1, the overall experiment was divided into conventional metaphors, familiarized metaphors, and control groups. For example, the metaphorical meaning of the prime “历史是镜子 /History is a mirror” is that we can learn from history, stay alert, and prevent repeating the same accident. Thus, its MT, LT, and UT were set to “反思 /introspection,” “梳妆 /makeup,” and “水杯 /cup,” respectively.

**Table 1:** Sample stimuli in the ERP study.

	**Prime**	**Probe**		
Conventional metaphor	历史是镜子 (History is a mirror)	MT 反思 (introspection)	LT 梳妆 (makeup)	UT 水杯 (cup)
	雪花是羽毛 (Snowflake is feather)	Non-word 先舒		
Familiarized metaphor	态度是迷雾 (Attitude is fog)	MT 朦胧 (elusiveness)	LT 水汽 (moisture)	UT 木头 (wood)
	才华是光芒 (Talent is light)	Non-word 格班		
Control sentence	玫瑰是鲜花 (Rose is flower)	Word 种子 (seed)		
	地图是工具 (Map is tool)	Non-word 笔已		

For the rigor of the experimental materials, the final 80 metaphors in this study were screened from 260 sentences, and the criteria were familiarity, acceptability, aptness, and word frequency. The original 260 Chinese metaphors in the form of “X is (a) Y” were largely based on some related studies (Lakoff and Johnson, 1980; Pynte et al., 1996; Sam and Catrinel, 2006; Wang et al., 2019; Sun et al., 2022) with the vehicle always in the last position without any variation. The criteria and specific process for the final selection from 260 to 80 metaphors were as follows.

Notably, familiarity, acceptability, and aptness are important for metaphor processing. Typically, familiarity is the index to judge whether a given metaphor is conventional or novel (Lai et al., 2009; Lai and Curran, 2013). Acceptability refers to the subjective sense of whether the given expression is acceptable to the participants (Lai et al., 2009; Lai and Curran, 2013). In addition, the reason for measuring aptness was that aptness might play a role in understanding metaphors (Sam and Catrinel, 2006) since novel but apt metaphors could be accessed directly. The control of aptness was to ensure that the metaphoricity of the materials was all highly appropriate.

First, thirty participants from Tsinghua University who did not participate in the ERP experiment rated these 260 randomly ordered metaphorical phrases on aptness (1 = very highly unapt and 7 = very highly apt). Metaphors averaged scores lower than 4 were deleted for lack of metaphoricity. Next, another sixty participants were separated into two groups (each with thirty participants) and asked to rate the remaining metaphors on familiarity (1 = very highly unfamiliar and 7 = very highly familiar) and acceptability (1 = very highly unacceptable and 7 = very highly acceptable), respectively. Finally, forty conventional metaphors (with higher scores in familiarity) and forty familiarized metaphors (with lower scores in familiarity) were selected as experimental metaphors (conventional vs. familiarized = 5.78 vs. 2.98, *p* < 0.001); the acceptability for conventional metaphors was higher than familiarized metaphors as previous studies did (Lai et al., 2009; Lai and Curran, 2013), and the aptness was comparable (conventional vs. familiarized = 4.3 vs. 4.4, *p* > 0.05) between the two conditions.

In addition, to exclude the effect of frequency, as it is an important factor in language studies (Bybee, 2006, 2010; Norris, 2013), we calculated the frequency of the final word of the 80 metaphors from the Xiandai Hanyu Pinlu Cidian [Modern Chinese Frequency Dictionary], and there was no difference between conventional (*M* = 338, SD = 59.1) and familiarized metaphors (*M* = 329, SD = 25.6), which indicated that it is the participants' subjective familiarity with the co-occurrence of the topic and vehicle of the metaphors that determined conventionality, and reassure the validity of the selected materials. All the selected metaphors were literally incongruous. Since metaphors are non-literal expressions, participants were supposed to access the non-literal/metaphorical meaning.

Finally, the formal MT, LT, and UT in the 80 metaphors were, in turn, strictly controlled in terms of three dimensions: semantic relatedness, word frequency, and concreteness, respectively. Formal eighty metaphors paired with MT or UT formed a list to be rated from 1 to 5 on semantic relatedness. Each metaphor is paired with two candidate MTs (for example, “History is a mirror” was paired with “retrospection” and “introspection” respectively) to determine the more suitable MT and paired with one UT (for example, “History is a mirror” was paired with “cup”) as control. The total number of word pairs to be rated was 240, and the order was randomized before being rated. A similar design was also conducted in the eighty vehicles paired with LT or UT. All probe words were two characters, ensuring the same word length. A total of 60 students from Tsinghua University who did not participate in the ERP experiment nor the former rating were paid to rate these pairs, with each scale evaluated by 30 participants. The higher scores of candidates using the same metaphor were selected as the experimental material. And the higher scores of eighty LT and eighty MT were chosen. LT was rated significantly higher than UT (*p* < 0.001), and MT was rated significantly higher than UT (*p* < 0.001). The scores for MT showed no difference between conventional (*M* = 4.28, SD = 0.31) and familiarized metaphors (*M* = 3.86, SD = 0.33), and neither did LT. The mean score was 4.07 (SD = 0.38) for the association between the MT and metaphor and 4.13 (SD = 0.45) between the LT and vehicle. There was no difference between the two means (*p* > 0.05).

Word frequency was also calculated for these formal probe words. MT showed no difference between conventional (*M* = 392, SD = 74.3) and familiarized metaphors (*M* = 453, SD = 78.7), and frequency for LT also showed no difference between conventional (*M* = 260, SD = 24.7) and familiarized metaphors (*M* = 320, SD = 37.6). Similarly, frequency for UT showed no difference between conventional (*M* = 337, SD = 44.3) and familiarized metaphors (*M* = 383, SD = 51.5), which excluded the effect of frequency. In addition, we also distributed concreteness questionnaires, including all formal probe words, to a group of 30 students on a 7-point Likert scale (1 = very abstract and 7 = very concrete). Results showed that MT, LT, and UT were significantly different from each other. The average score for MT (*M* = 3.09, SD = 0.56) was significantly lower than LT (*M* = 4.67, SD = 0.97, *p* < 0.05) and UT (*M* = 5.03, SD = 1.13, *p* < 0.05), and the score for LT was significantly lower than UT (*p* < 0.05). For familiarized metaphor prime, MT (*M* = 3.14, SD = 0.59) was significantly lower than LT (*M* = 4.46, SD = 0.93, *p* < 0.05) and UT (*M* = 5.05, SD = 1.1, *p* < 0.05), and the score for LT was significantly lower than UT (*p* < 0.05). For the conventional metaphor prime, MT (*M* = 3.03, SD = 0.53) was significantly lower than LT (*M* = 4.88, SD = 0.97, *p* < 0.05) and UT (*M* = 5.02, SD = 1.18, *p* < 0.05), and the score for LT showed no difference than that for UT.

The final 720 pairs were split into four blocks. Each block contained 60 conventional metaphors, 60 familiarized metaphors, and 60 control primes, each with a half-word and a half-non-word probe. The order of item presentation within each block was randomized, and the block order was counterbalanced across the participants. To minimize repetitive effects, the same prime sentence appeared only once in a block. In addition, no repeated word was used in any trial and in the same block in order to further decrease the effect of repetition.

### 2.3 Procedure

**Figure 1:**
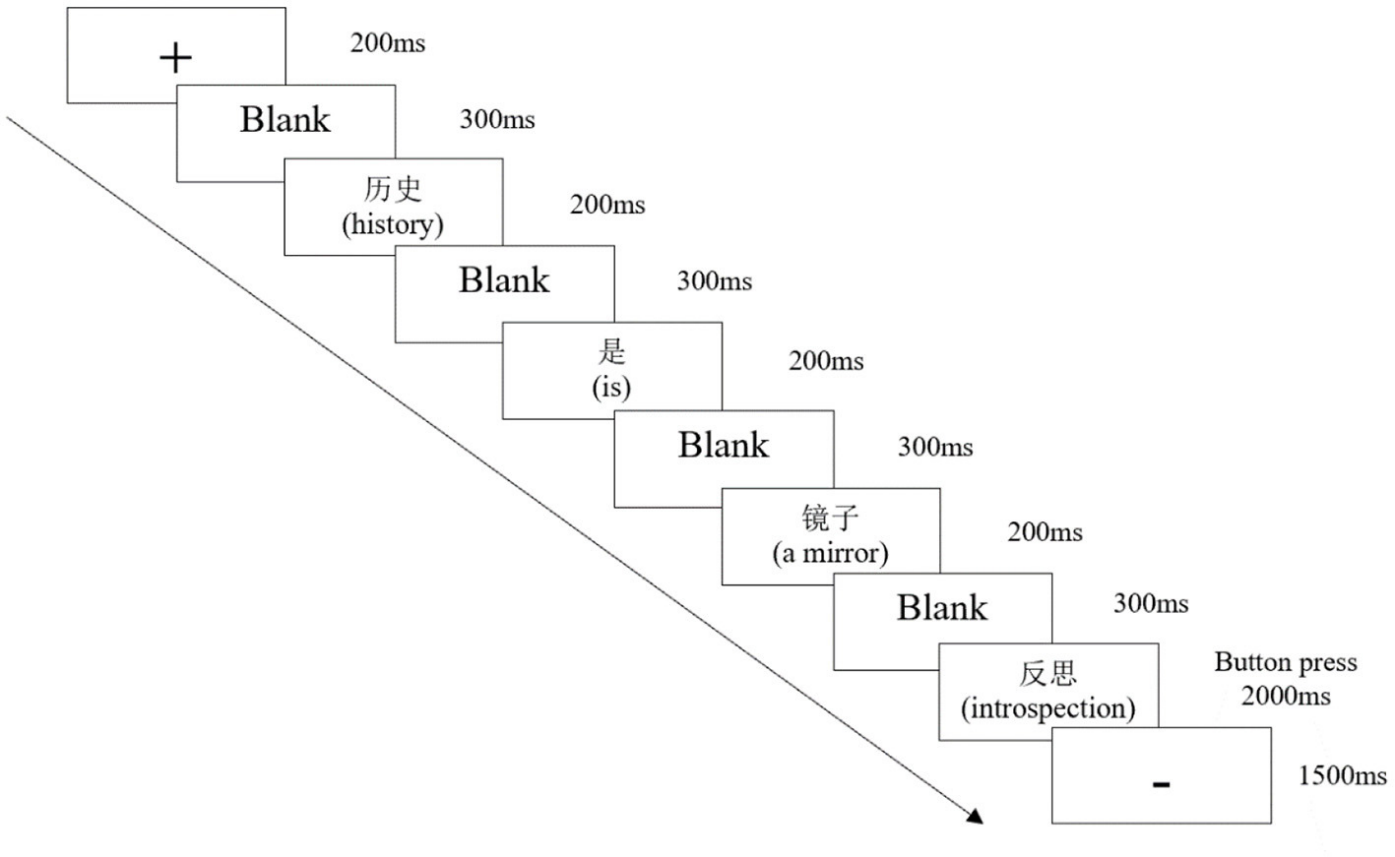
Experimental procedure.

Participants were seated in a dimly lit, sound-attenuated room and were instructed to focus on the middle of the screen and avoid body movements and eye blinks. All stimuli were presented word-by-word on an LED computer screen 80 cm from the participants. We partially replicated Coulson and Van Petten (2002), Lai et al. (2009), and Lai and Curran (2013) regarding stimulus presentation. Each word in the sentence was presented for 200 ms with an inter-word interval of 300 ms. After the end word of the sentence appeared and a blank interval of 300 ms, the probe word presented 2,000 ms. Participants judged whether the probe was a word or non-word based on comprehending the prime sentence by pressing one of two buttons. After pressing the button or no response was detected within 2,000 ms, “–” appeared for 1,500 ms for the participants to blink. The experimental procedure is illustrated in Figure 1. All participants were informed that three words made up the prime sentence, the fourth word (probe) was independent of the Prime, and they needed to judge whether it was a word. Each participant judged 30 trials for practice, and only when the accuracy reached 80% could the formal experiment begin. Participants could take a 2–5 min break between blocks. The experiment, including the electrode preparation, lasted ~2 h.

After the formal experiment, a *post-hoc* test was conducted to ensure each participant paid attention to prime sentences. A total of 30% of the formal experiment trials were extracted, and an equal number of sentences that did not appear in the experiment were designed. Sentences were presented to participants after the EEG test. They were asked to judge whether they had seen these sentences in the experiment. We designed this to ensure that participants paid attention to the Prime and processed these sentences, which is also consistent with Rubio Fernandez (2007). Only a correction rate of over 66% could be used (Blasko and Connine, 1993).

### 2.4 EEG recording and analysis

Electroencephalograms (EEG) were recorded from 62 Ag/AgCl electrodes in an elastic cap configured using the international 10–20 electrode placement system. The vertical electrooculogram (VEOG) was recorded using electrodes below the left eye, and the horizontal electrooculogram (HEOG) was recorded using electrodes at the outer canthus of the right eye. All electrode impedances were kept below 10 kΩ. The EEG signals were amplified using a BrainAmp DC amplifier system with a bandpass of 0.01–100 Hz and continuously sampled at 500 Hz (Sun et al., 2022). Trials were discarded from analyses if more than 20% of channels were bad (average amplitude over 100 μV or transit amplitude over 50 μV).

All electrodes were referenced offline to obtain the average. The ERP recording was time-locked to the onset of the word for each trial, and ERP analysis was applied to an epoch extending from 200 ms before to 1,000 ms after onset. Semiautomatic ocular correction with independent component analysis was performed. The EEGs were bandpass filtered offline from 0.05 to 30 Hz (zero phase shift mode, 24 dB/oct). Epochs exceeding ±80 μV were automatically discarded by artifact rejection, and trials that responded incorrectly were eliminated.

Two-time windows were selected: 300–500 ms (Tartter et al., 2002; Lai and Curran, 2013; Forgács et al., 2015) and 550–800 ms (Goldstein et al., 2012) for N400 and P600 components, respectively. A repeated measures analysis of variance (ANOVA) was performed on the average amplitude of each time window. The three within-participant factors were prime type (conventional and familiarized), probe word type (MT, LT, and UT), and lateral areas or midline electrodes (Fz, Cz, and Pz). The laterality and anteriority factors were crossed, forming six lateral regions of interest (ROI) and each region has 5–6 representative electrodes: left anterior/LA (F3, F5, F7, FC3, FC5, and FT7); left central/LC (C3, C5, T7, CP3, CP5, and TP7); left posterior/LP (P3, P5, P7, PO3, and PO7); right anterior/RA (F4, F6, F8, FC4, FC6, and FT8); right central/RC (C4, C6, T8, CP4, CP6, and TP8); and right posterior/RP (P4, P6, P8, PO4, and PO8). Average mean ERP amplitude for each ROI over electrodes in each region. Greenhouse–Geisser correction was applied where appropriate. In these cases, we reported the uncorrected degrees of freedom and the corrected *p*-values. The effect sizes were presented as partial eta-squared (η^2^_*p*_) for the F-tests. RTs < 250 ms and >1,750 ms were excluded from all analyses (Blasko and Connine, 1993). Only correct responses were entered into the analysis.

## 3 Results

### 3.1 Behavioral results

**Figure 2:**
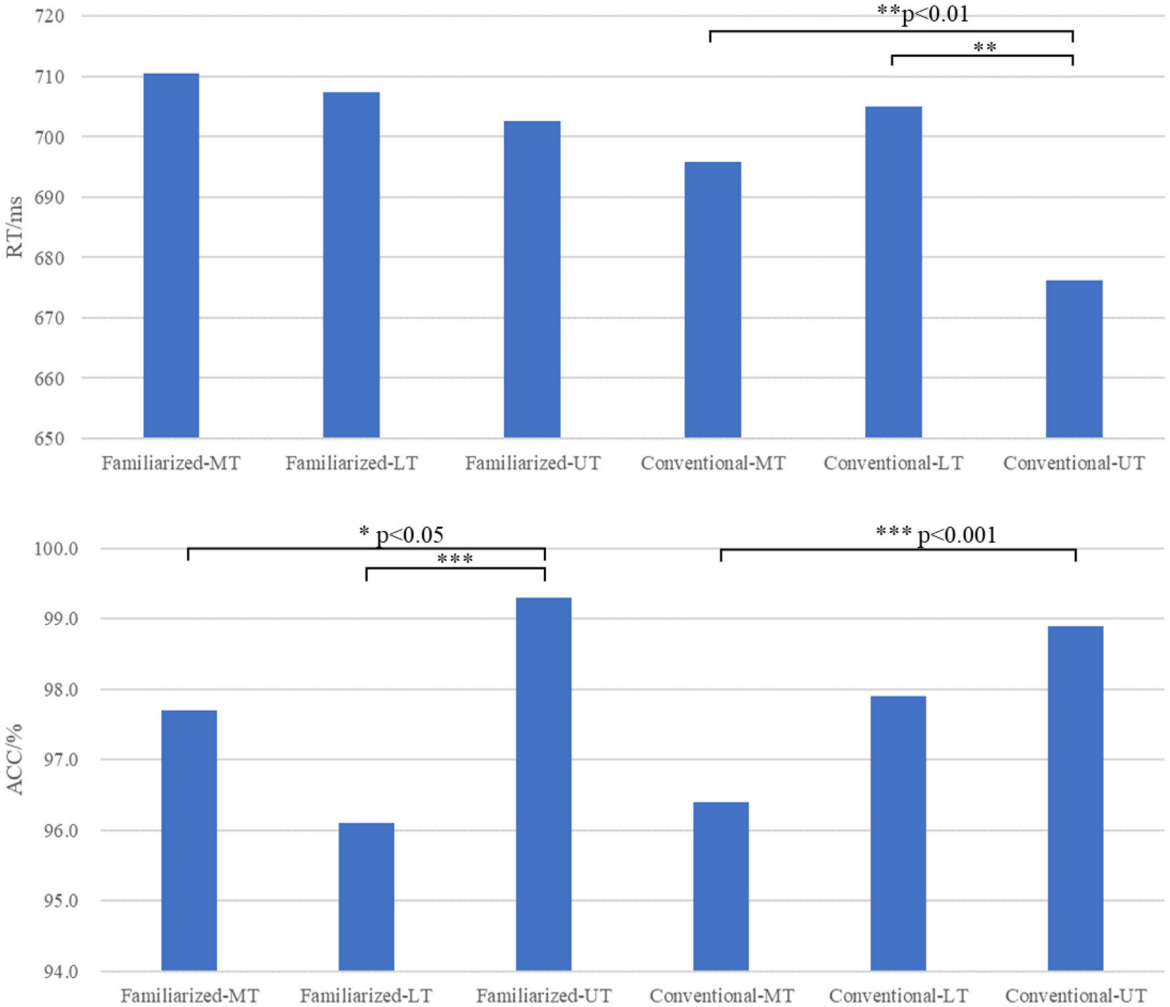
RT and ACC of MT, LT, and UT in familiarized and conventional metaphor primes.

Figure 2 shows the mean RT and ACC for MT, LT, and UT responses in conventional and familiarized metaphors. The grand mean accuracy rate for the lexical decision task for the experimental sentences was 97.9% (SD = 0.142). The high level of accuracy indicated that participants fully understood the requirements of the experiment and paid attention to the stimuli.

A 2 prime (familiarized /conventional) × 3 probe (MT/LT/UT) repeated measures ANOVA was conducted. The analysis of RT indicated a significant main effect for both prime [*F*_(1, 24)_ = 10.489, *p* = 0.003, $\eta_{p}^{2}$ = 0.304] and probe [*F*_(2, 48)_ = 4,734, *p* = 0.013, $\eta_{p}^{2}$ = 0.165], with no interaction [*F*_(1, 24)_ = 2.270, *p* = 0.114, $\eta_{p}^{2}$ = 0.086]. The RT for familiarized metaphors was significantly longer than that for conventional metaphors (conventional vs. familiarized = 692.393 ms vs. 706.907 ms, *p* = 0.003), and the RT for MT and LT was significantly longer than that for UT (MT vs. UT = 703.267 ms vs. 689.437 ms, *p* = 0.032; LT vs. UT = 706.245 ms vs. 689.437 ms, *p* = 0.036). No difference was found between MT and LT (MT vs. LT = 703.267 ms vs. 706.245 ms, *p* > 0.05). Regarding ACC, the result revealed a main effect of Probe [*F*_(2, 48)_ = 12.245, *p* < 0.001, $\eta_{p}^{2}$ = 0.338], interacted with prime [*F*_(2, 48)_ = 8.774, *p* = 0.001, $\eta_{p}^{2}$ = 0.268]. *Post-hoc* mean comparisons with Bonferroni showed that under familiarized metaphor prime, ACC of UT was significantly higher than that of MT (UT vs. MT = 0.993 vs. 0.977, *p* = 0.02) and LT (UT vs. LT = 0.993 vs. 0.961, *p* < 0.001). No difference was found between MT and LT (MT vs. LT = 0.977 vs. 0.961, *p* = 0.06). Under the conventional metaphor prime, ACC of UT was significantly higher than MT (UT vs. MT = 0.989 vs. 0.964, *p* < 0.001), and no difference was found between MT and LT (MT vs. LT = 0.964 vs. 0.979, *p* > 0.05) and between LT and UT (LT vs. UT = 0.979 vs. 0.989, *p* > 0.05).

### 3.2 Event-related potentials

**Figure 3:**
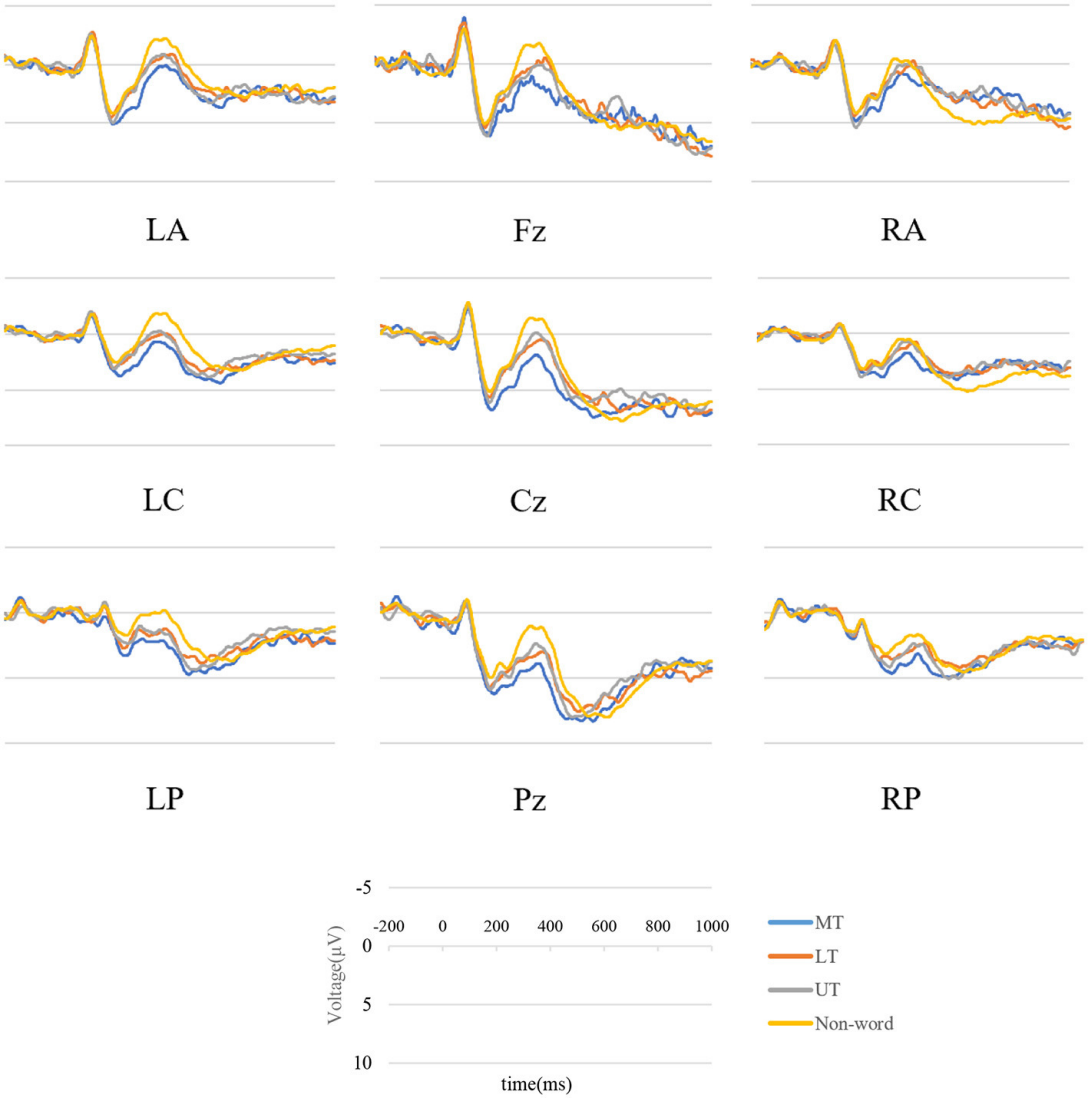
Grand average ERP waveforms in nine groups under familiarized metaphor prime.

**Figure 4:**
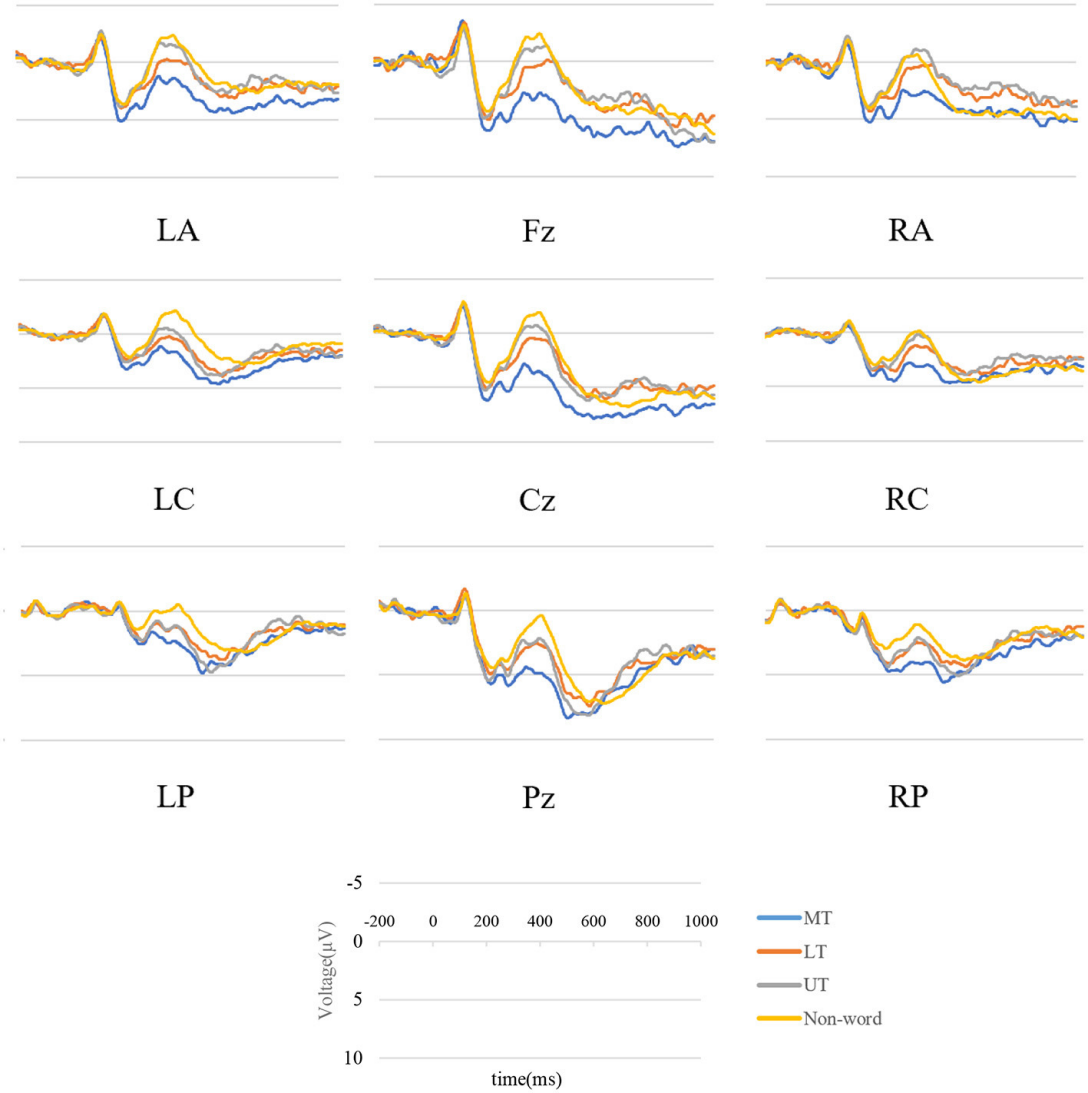
Grand average ERP waveforms in nine groups under conventional metaphor prime.

**Figure 5:**
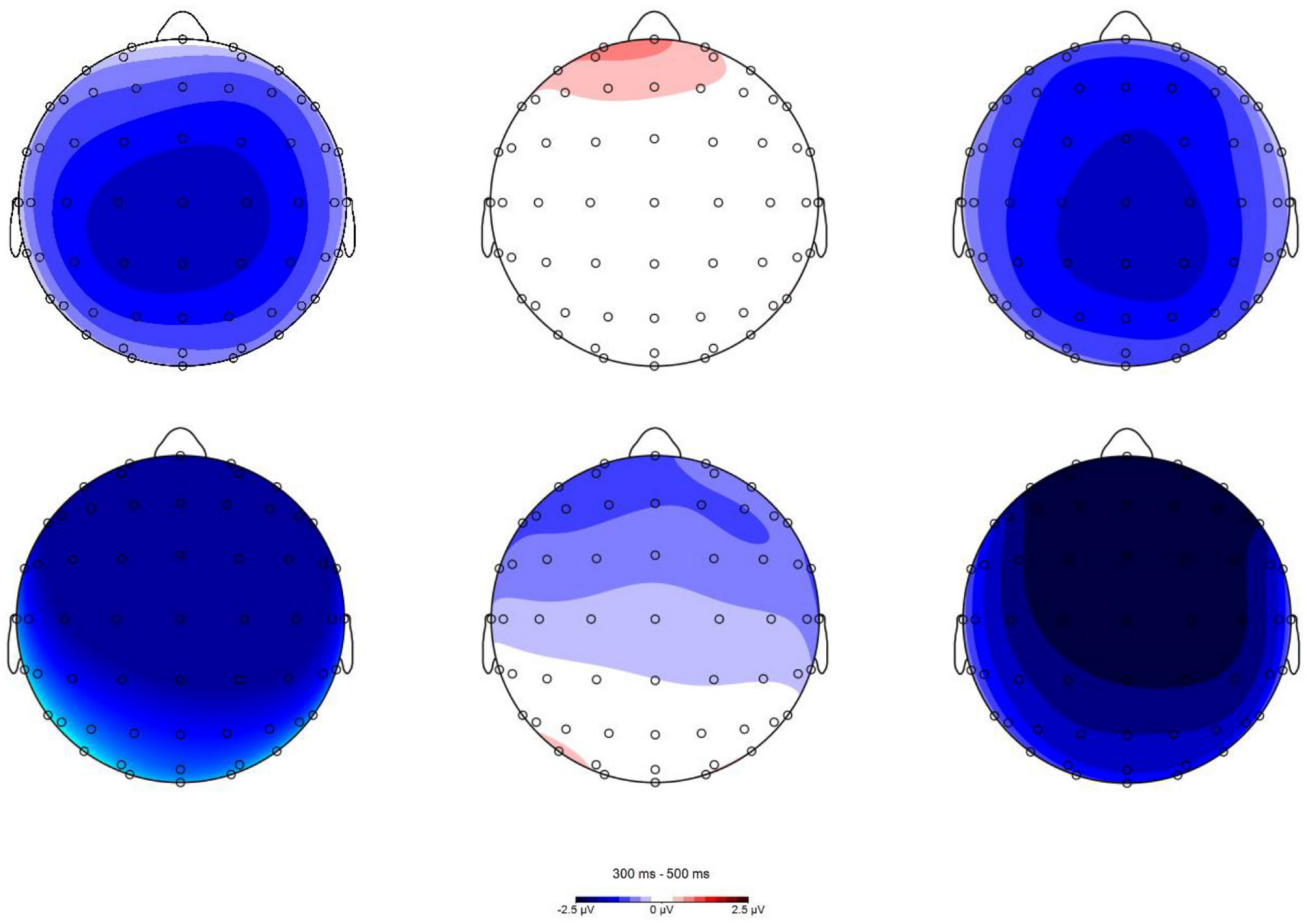
Topographic plots of UT-MT, UT-LT, and LT-MT differences in N400 (300–500 ms) time window in familiarized metaphor (the first row) and conventional metaphor prime (the second row).

**Figure 6:**
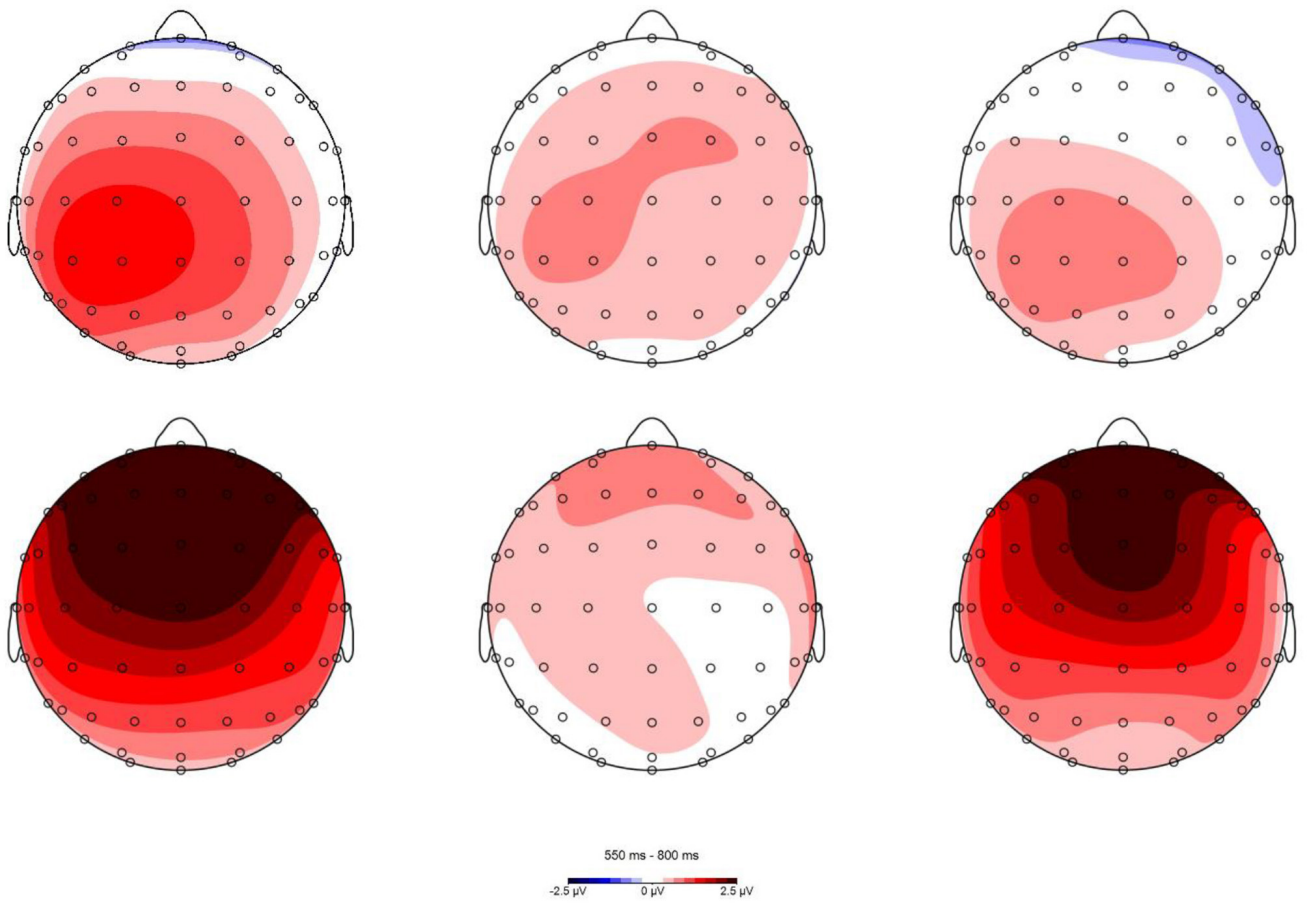
Topographic plots of MT-UT, LT-UT, and MT-LT differences in P600 (550–800 ms) time window in familiarized metaphor (the first row) and conventional metaphor prime (the second row).

Visual inspection and data analysis predefined the time windows of interest for electrophysiological scalp data. The amplitude measurements were based on the average amplitude within specified time windows. The mean amplitudes of the probe words were extracted from 300–500 ms (N400) and 550–800 ms (P600). Grand averaged waveforms for the N400 and P600 for the four probe types and two primes are displayed in Figures 3, 4. The topographical maps of the differential waves in response to the familiarized and conventional primes are shown in Figures 5, 6. We mainly focused on the N400 under metaphor primes and briefly analyzed control primes and the P600.

Repeated measurement analysis of variance was conducted on the metaphor and control primes with UT in N400 and P600 to compare the differences between metaphor and control primes. The results showed that the prime sentence type's main effect was insignificant, indicating that UT showed no difference in different primes, and the results and analysis below only focused on metaphor primes, which is also the main focus of this study. A 2 prime × 3 target × 9 location repeated measures ANOVA was conducted in the N400 and P600 time windows.

#### 3.2.1 N400

The results yielded a main effect of Probe [*F*_(2, 48)_ = 21.840, *p* < 0.001, $\eta_{p}^{2}$ = 0.476] and Location [*F*_(8, 192)_ = 10.757, *p* < 0.001, $\eta_{p}^{2}$ = 0.309], with interaction [*F*_(16, 384)_ = 4.316, *p* < 0.001, $\eta_{p}^{2}$ = 0.152], and a Prime × Probe × Location interaction [*F*_(16, 384)_ = 2.592, *p* < 0.001, $\eta_{p}^{2}$ = 0.097]. The interaction was then broken down by Prime. For the familiarized metaphor prime, the results showed a significant main effect of Probe [*F*_(2, 48)_ = 4.413, *p* = 0.017, η^2^_*p*_ = 0.155] and Location [*F*_(8, 192)_ = 11.182, *p* < 0.001, $\eta_{p}^{2}$ = 0.318] with no interaction [*F*_(16, 384)_ = 0.854, *p* = 0.510, $\eta_{p}^{2}$ = 0.034]. LT elicited significantly more negative N400 than MT (LT vs. MT:1.852 μV vs. 2.917 μV, *p* < 0.05), UT elicited significantly more negative N400 than MT (UT vs. MT:1.991 μV vs. 2.917 μV, *p* < 0.05). No difference was found between LT and UT (*p* > 0.05). Midline and posterior electrodes elicited significantly larger amplitude than other electrodes (*p*s < 0.05). For the conventional metaphor prime, results showed the main effect of Probe [*F*_(2, 48)_ = 20.142, *p* < 0.001, $\eta_{p}^{2}$ = 0.456] and Location [*F*_(8, 192)_ = 9.714, *p* < 0.001, $\eta_{p}^{2}$ = 0.288], with interaction [*F*_(16, 384)_ = 6.802, *p* < 0.001, $\eta_{p}^{2}$ = 0.221]. When it was further broken down, midline electrodes elicited significantly larger amplitude than lateral electrodes in MT, LT, and UT (*p*s < 0.05). In any electrode, MT elicited significantly less negative N400 than LT and UT (*p*s < 0.05), and no difference was found between LT and UT in all electrodes (*p*s > 0.05).

Considering the significant difference in concreteness ratings may affect N400, an item-based ANOVA analysis was conducted with concreteness as a covariate. Results revealed that there was a significant effect of concreteness [*F*_(1, 1923)_ = 17.189, *p* < 0.001, $\eta_{p}^{2}$ = 0.009], Probe [*F*_(2, 1923)_ = 12.757, *p* < 0.001, $\eta_{p}^{2}$ = 0.013], Location [*F*_(8, 1923)_ = 34.107, *p* < 0.001, $\eta_{p}^{2}$ = 0.142], and an interaction between Prime and concreteness [*F*_(1, 1923)_ = 4.092, *p* = 0.04, $\eta_{p}^{2}$ = 0.002]. No interaction was found between Probe and concreteness [*F*_(2, 1923)_ = 0.995, *p* = 0.370, $\eta_{p}^{2}$ = 0.001], nor in Prime × Probe × concreteness [*F*_(2, 1923)_ = 0.203, *p* = 0.817, $\eta_{p}^{2}$ = 0.0002], nor in Prime × Probe × Location × concreteness [*F*_(16, 1923)_ = 0.823, *p* = 0.66, $\eta_{p}^{2}$ = 0.006]. The Prime × concreteness interaction was broken down by Prime. For the familiarized metaphor prime, the results showed a significant main effect of Probe [*F*_(2, 948)_ = 3.155, *p* = 0.04, $\eta_{p}^{2}$ = 0.006] and Location [*F*_(8, 948)_ = 14.215, *p* < 0.001, $\eta_{p}^{2}$ = 0.120] with no interaction [*F*_(16, 948)_ = 0.598, *p* = 0.887, $\eta_{p}^{2}$ = 0.010]. LT elicited significantly more negative N400 than MT (LT vs. MT = 0.642 μV vs. 1.012 μV, *p* = 0.03), UT elicited significantly more negative N400 than MT (UT vs. MT = 0.636 μV vs. 1.012 μV, *p* = 0.03), no difference was found between LT and UT (LT vs. UT = 0.642 μV vs. 0.636 μV, *p* > 0.05). Posterior electrodes elicited significantly larger amplitude than frontal electrodes (*p*s < 0.05). For the conventional metaphor prime, the results showed a significant main effect of Probe [*F*_(2, 975)_ = 11.148, *p* < 0.001, $\eta_{p}^{2}$ = 0.023], Location [*F*_(8, 975)_ = 21.073, *p* < 0.001, $\eta_{p}^{2}$ = 0.173], and concreteness [*F*_(1, 975)_ = 20.494, *p* < 0.001, $\eta_{p}^{2}$ = 0.021], with no interaction [*F*_(16, 975)_ = 0.626, *p* = 0.864, $\eta_{p}^{2}$ = 0.010]. LT elicited significantly more negative N400 than MT (LT vs. MT = 0.483 μV vs. 1.036 μV, *p* = 0.001), UT elicited significantly more negative N400 than MT (UT vs. MT = 0.339 μV vs. 1.036 μV, *p* < 0.001), no difference was found between LT and UT (LT vs. UT = 0.483 μV vs. 0.339 μV, *p* > 0.05). Posterior electrodes elicited significantly larger amplitude than frontal electrodes (*p*s < 0.05).

#### 3.2.2 P600

The result yielded a main effect of Probe [*F*_(2, 48)_ = 4.235, *p* = 0.02, $\eta_{p}^{2}$ = 0.150], interacted with Prime and Location [*F*_(16, 384)_ = 2.592, *p* = 0.041, $\eta_{p}^{2}$ = 0.097]. The interaction was then broken down by Prime. In the familiarized metaphor prime, Location showed a significant main effect [*F*_(8, 192)_ = 11.035, *p* < 0.001, $\eta_{p}^{2}$ = 0.315], neither probe [*F*_(2, 48)_ = 0.418, *p* = 0.661, $\eta_{p}^{2}$ = 0.017] nor interactions [*F*_(16, 384)_ = 0.868, *p* = 0.501, $\eta_{p}^{2}$ = 0.035] were significant. Midline electrodes elicited significantly more positive waveforms than lateral electrodes (*p*s < 0.05). In the conventional metaphor prime, the results showed a main effect of the probe [*F*_(2, 48)_ = 5.491, *p* = 0.007, $\eta_{p}^{2}$ = 0.186] and location [*F*_(8, 192)_ = 10.543, *p* < 0.001, $\eta_{p}^{2}$ = 0.305], with interaction [*F*_(16, 384)_ = 4.851, *p* < 0.001, $\eta_{p}^{2}$ = 0.131]. When it was further broken down, midline electrodes elicited significantly greater amplitude than lateral electrodes in MT, LT, and UT (*p*s < 0.05). MT elicited significantly more positive P600 than UT in central and frontal electrodes (*p*s < 0.05), and no difference was found among MT, LT, and UT in posterior electrodes (*p*s > 0.05).

## 4 Discussion

This study aimed to figure out whether conventional and familiarized metaphors have the same processing mechanism and to what extent metaphorical meaning is accessed in the late stage of metaphor processing. Three main probes were designed following conventional and familiarized metaphor primes, and a specific time span (300 ms) was set to examine meaning access, which was recorded and analyzed by ERPs for the first time. That is, the way we speculate the processing for metaphors is by presenting probe words following them instead of examining the metaphors themselves. In general, our results showed that LT elicited significantly more negative N400 than MT in both primes, and there was no difference between LT and UT. Regarding the reaction time, RT for LT and MT was significantly longer than UT. Notably, LT has a longer reaction time and a more negative N400, whereas MT has a longer reaction time and a less negative N400. In P600, there was no difference between MT and LT in the conventional and familiarized metaphors. Based on these findings, we hypothesize that both processing conventional and familiarized metaphors support the direct processing model to some extent.

One of the main findings was the results about MT and LT in N400. In the familiarized metaphor Prime, LT elicited a more negative waveform than MT. In the conventional metaphor prime, LT and UT elicited more negative waveforms than MT. MT triggered the least waveform in both familiarized and conventional primes, suggesting the metaphorical meaning of both primes has been accessed 500 ms after the onset of its last word (late stage of sentence processing). Specifically, MT was pre-activated before being presented, and less effort is required to access the semantic information of the probe, reducing the amplitude of N400.

Notably, no difference was found when comparing MT between familiarized and conventional metaphor primes. LT also showed no difference in both primes, suggesting that the metaphorical meaning of both conventional and familiarized metaphors was accessed directly. This finding was partially similar to that by Blasko and Connine (1993). They examined metaphor processing using a cross-modal priming paradigm and found that MT for novel metaphors with high aptness was available as fast as conventional metaphors, suggesting that the figurative interpretation of a low familiar metaphor could become available immediately (ISI = 0 ms) after the offset of the metaphor's vehicle if the metaphor was highly apt. Their finding was consistent with our results, and both supported the direct model (Glucksberg and Keysar, 1990). However, there are also differences between the two studies: (1) metaphors in their study were presented in mid-sentences [e.g., Prime: The belief that HARD WORK IS A LADDER is common to this generation. Target: advance (MT)/ rungs (LT)/pastry (UT)], whereas we presented the metaphor independently. (2) They used a cross-modal priming paradigm (earphones acoustically presented primes and screen visually presented targets, which Al-Azary and Katz, 2021 and Rubio Fernandez, 2007 also applied), whereas we only used visual presentation. (3) They reported the immediate priming for MT and LT after prime onset (ISI = 0 ms) in conventional and novel but highly apt metaphors. We found that MT was accessed 500 ms (ISI = 300 ms, SOA = 500 ms) after the vehicle onset for both primes, but we both suggested that metaphorical meaning could be accessed directly.

The results of this study supporting the direct processing model in both the conventional and familiarized metaphors may also be related to aptness. Recall that there was no difference between conventional and familiarized metaphors in aptness ratings in our study, scores both averaged >4, which means that even familiarized metaphors were comprehensive. As Sam and Catrinel (2006) and Glucksberg (2008) suggested, novel metaphors with high aptness could be understood as readily as conventional metaphors. Thus, high aptness may effectively facilitate the processing of conventional and familiarized metaphors in our study.

In addition, the result that MT elicited significantly less negative N400 than LT, and there was no difference between LT and UT, suggesting that the literal meaning of the Prime was suppressed 500 ms after the onset of the vehicle. Moreover, the reaction time (RT) for LT also supported this speculation. Our results revealed that the RT of LT is significantly longer than UT. No priming should yield higher N400 and slower RT. Thus, it is possible that LT was not primed. This result contradicts some previous studies (Rubio Fernandez, 2007; Al-Azary and Katz, 2021). In Rubio Fernandez (2007) study, three kinds of ISI (0/400/1,000 ms) were constructed to examine meaning suppression in metaphor. Superordinates (similar to LT), distinctive properties (similar to MT), control words, and non-words were targets followed by novel metaphor (Prime). The results showed that both superordinates and distinctive properties were primed in the ISI of 0 and 400 ms, whereas superordinates were suppressed at the ISI of 1,000 ms. In other words, superordinates were activated up to 400 ms after prime onset and then suppressed between 400 and 1,000 ms. Our study showed no priming for LT (ISI = 300 ms), even for familiarized metaphors, suggesting that the literal meaning may be suppressed 500 ms after the onset of the vehicle. This conflict may be due to the length of the context or the cross-modal priming paradigm. Nevertheless, we both supported the direct model (Glucksberg and Keysar, 1990) to some extent since our studies both revealed that literal meaning need not be accessed and rejected before metaphorical meaning was accessed; that is, metaphorical meaning could be accessed directly. Similarly, Glucksberg and Keysar (1990) suggested that metaphor interpretation involved enhancing metaphor-relevant properties of the vehicle while actively suppressing metaphor-irrelevant ones. In two eye-tracking experiments, Ronderos et al. (2021) also provided evidence that features essential for understanding the meaning of critical words but are not part of the metaphoric interpretation are ignored during the construction of metaphorical meaning.

Regarding the speculation of LT, our results also conflict with Al-Azary and Katz (2021). In their study, novel metaphors (e.g., highways are snakes) primed bodily action associates (i.e., slither, similar to UT) but not abstraction associates (i.e., danger, similar to MT), whereas conventional metaphors primed abstraction associates but not bodily action associates in both 0 ms and 1,000 ms ISI. That is, familiarity determines different processing mechanisms. Novel metaphors only activated bodily action associates but not abstraction associates throughout, which was inconsistent with Glucksberg et al. (2001) and Rubio Fernandez (2007), and our study. As Al-Azary and Katz (2021) suggested, the associates were not used to capture the metaphor's meaning but to serve as proxies for simulation and abstraction processes. We speculate that the experimental aim and materials the researchers set and used may affect results. We set different probes to measure the meaning process for metaphor, whereas Al-Azary and Katz (2021) intended to examine simulation and abstraction instead of exploring specific meaning access. In addition, the target used by Al-Azary and Katz (2021) was not as similar as our study. The bodily action associates were not equal to our LT since LT was related to the literal meaning of vehicle but not limited to bodily action, which may cause different results between the two studies.

Our RTs and ERPs for LT are actually in agreement. The curious finding is the MT: these probes seem to be concurrently primed (low N400) and not primed (RT). The overall results of the behavioral data showed that RT for familiarized metaphor primes was significantly longer than conventional metaphor primes, which may indicate that familiarized metaphors were more difficult to process than conventional metaphors, consistent with most studies (e.g., Blasko and Connine, 1993; Arzouan et al., 2007). However, there was a contradiction between MT's reaction time and its N400. Our results revealed that RT for MT is significantly longer than UT, whereas MT elicited significantly less negative N400. It is generally agreed that targets with a more immediate response were supposed to elicit less negative N400, especially in ERP studies in word recognition (Wang et al., 2021, 2024). To identify whether it is an accidental phenomenon, we repeated our experiment with behavioral recording (see NOTE 1^1^), and the inconsistency was replicated. Both experiments showed a significantly longer reaction time for MT and LT than UT, which illustrated that the result may not be accidental.

Using a similar paradigm, we found that Sun et al. (2022) did not show the inconsistency. Specifically, they reported consistent results that MT elicited larger N400 and longer RT. The overall results of their behavioral data showed that RT for literary metaphor (novel metaphor) was longer than for non-literary metaphor (conventional metaphor), which is consistent with our finding. However, probes in their novel metaphor condition induced significantly negative N400 and longer response times than their literal condition. Their results reported an agreement between RT and ERP that contradicts our results. We speculate that the conflict between the two studies may stem from differences in materials, experimental design, and so on. First, the representation of metaphors in the two studies was different. Our metaphorical structure is “X is (a) Y” and has a fixed number of words, whereas their metaphors did not have a fixed structure or number of words. Second, the presentation of primes was different between the two studies. We presented prime word by word and set a specific time span to measure meaning access, whereas they presented prime one at a time for 2,500 ms without concerning specific time for meaning access. Third, our study used a lexical decision task, as do most metaphor studies using the prime-target paradigm (Blasko and Connine, 1993; Rubio Fernandez, 2007; Klepousniotou et al., 2012; Al-Azary and Katz, 2021), whereas Sun et al. (2022) operated a semantic relatedness task. All the differences in experimental design between the two studies could have led to different results. However, regarding topographical distribution, we found that N400 in both studies elicited the largest waveforms, mainly in the central areas (as shown in Figure 5).

However, the phenomenon of inconsistency between RT and N400 did exist, as reported by Huang et al. (2021). In their study, four types of sentence conditions were designed: (1) a correct condition (critical word with correct semantic and syntactic information), (2) a doubly violated real-word condition (critical word with incorrect semantic and syntactic information), (3) an ordinary pseudoword condition (critical non-word with no semantic or syntactic information), and (4) a homophonic pseudoword condition (critical non-word with a homophonic clue guiding the participants to the correct information needed to comprehend the sentence). The critical word was embedded in the sentence. The results showed that RT for the control condition was longer than the other three anomalous conditions, whereas for N400, the control condition elicited significantly less negative waveforms. Our results revealed that RT for MT is significantly longer than UT, whereas MT elicited significantly less negative N400. Both studies showed an inconsistency in that longer reaction times and less negative N400 were triggered under a given experimental condition. We hypothesize that N400 in this study may represent lexical access (Arzouan et al., 2007; Goldstein et al., 2012; Huang et al., 2021). Specifically, N400 reduction may reflect context-facilitated access to stored memory representations (Brouwer et al., 2017; Delogu et al., 2019). In our study, following a complete sentence, there was a single-word probe, for which the figurative sense of the sentence was supposed to provide lexical priming, and it did so according to a reduced N400 but not in terms of RT. This phenomenon may occur when MT triggers a smaller N400 but longer response time. Since a figurative meaning could partially prime the MT, as revealed by the ERPs, but the entire word (with all its other, additional meanings, etc.) was needed to be retrieved, which took just as long in RTs as the LT.

In addition, the item-based analysis in N400 revealed that concreteness effect was significant, concrete words induced significantly more negative N400 than abstract words, which is consistent with the results of most previous studies (Holcomb et al., 1999; Huang et al., 2010; Barber et al., 2013). However, the concreteness effect did not change the previous N400 results. LT and UT still elicited significantly more negative N400 than MT in both conventional and familiarized metaphors, and there was no difference between LT and UT, a result that was not altered by the inclusion of concreteness. Thus, concreteness affected N400 to some extent, it may not be sufficient to explain the difference between MT and LT, UT. It was more likely due to the processing of the metaphors that caused the differences between different probes.

In some literature examining metaphor processing, studies of the concreteness effect focused on word pairs (e.g., velvety lake, and scary movie in Forgács et al., 2015), explored differences in the processing of metaphorical phrases vs. concrete and abstract phrases by moderating adjective concreteness, and found that the processing of metaphorical expressions was not strictly or merely driven by lexical or conceptual concreteness (Forgács et al., 2015), which is to some extent consistent with our findings. It should also be acknowledged that concreteness may be a factor influencing metaphor processing, and some studies have reported the concreteness effect of metaphors (Lai et al., 2019; Canal et al., 2022). Our study differs from the above research in that we reported the processes following the comprehension of a metaphor by way of presenting probe words instead of reporting ERPs directly related to the comprehension of metaphors, which may lead to concreteness playing a limited role in the present paradigm compared with that in previous studies. In the present study, the results were more likely due to the participants' activation and access to the metaphorical meanings.

In the P600 dimension, there was no difference between MT and LT in the conventional and familiarized metaphor primes. The functional significance of the ERP components N400 and P600 remains controversial. An important dimension of the two components may be in terms of automaticity vs. attentional control, with the N400 amplitude reflecting more automaticity and the P600 amplitude reflecting more control. In our study, the response times for both MT and LT were around 700 ms, which means that participants had already completed their judgments on the probe words before 700 ms, so more control processing may not be needed in the P600 time window. Regarding topographical distribution of P600, MT yielded a more positive waveform in frontal rather than posterior under conventional metaphor primes, with differences of distribution focused on the anterior and posterior but not between laterals, which is consistent with Coulson and Van Petten (2002). In general, the distribution is identical to our hypothesis, and the wide scalp activation entails the two hemispheres involved in metaphor processing (Klepousniotou et al., 2012).

Finally, we need to state that this study also has some drawbacks. First, our results showed that metaphorical meaning was accessed and literal meaning was suppressed in both primes, indicating that participants may understand metaphorical meanings for familiarized metaphors directly. There is also another explanation: participants possibly do not fully understand the Prime; the probe helps them access metaphorical meaning (Yang et al., 2013), as participants could find a close relationship between MT and its Prime. Considering this, further studies could explore metaphors independently to test this hypothesis. Second, metaphors take a variety of forms, and more flexible structures of metaphor could be examined in further studies.

In conclusion, this study examined Chinese metaphor processing by setting different probe words after metaphor primes. Results revealed suppressed literal meaning and enhanced metaphorical meaning, and no difference was found between conventional and familiarized metaphors, which brought additional evidence for understanding metaphorical processing mechanisms. In general, these findings provided new insights into measuring metaphor processing in an exact time window and possibly supported the direct processing model.

## Data availability statement

The raw data supporting the conclusions of this article will be made available by the authors, without undue reservation.

## Ethics statement

The studies involving humans were approved by the Ethics Committee of the School of Psychology at Tsinghua University. The studies were conducted in accordance with the local legislation and institutional requirements. The participants provided their written informed consent to participate in this study.

## Author contributions

XX: Writing – original draft, Validation, Methodology, Investigation, Formal analysis, Conceptualization. JZ: Writing – review & editing, Methodology, Investigation. YW: Validation, Methodology, Formal analysis, Data curation, Writing – review & editing, Conceptualization. MJ: Supervision, Project administration, Funding acquisition, Writing – review & editing, Conceptualization.

## Footnotes

^1^NOTE 1: Participants A total of 30 native Chinese students (mean age = 23 years, range = 18–25 years; 15 males) from Tsinghua University participated in this study. All the participants were right-handed with normal or corrected-to-normal vision before the experiment. Handedness was assessed by the Edinburgh Handedness Survey (Oldfield, 1971). None of them reported a history of neurological or psychiatric impairments. Informed consent was obtained from all the participants following the guidelines of the Declaration of Helsinki. Stimuli All experimental stimuli were identical to the former ERP study. Procedure All procedures were identical to the ERP study except the EEG recording and analysis. Results The grand mean accuracy rate for the lexical decision task for the experimental sentences was 97.2% (SD = 0.03), indicating that participants fully understood the requirements of the experiment and paid attention to the stimuli. A 2 prime (familiarized /conventional) × 3 probe (MT/LT/UT) repeated measures ANOVA was conducted. The analysis of RT indicated a significant main effect for Probe [*F*_(2, 58)_ = 14.968, *p* < 0.001, $\eta_{p}^{2}$ = 0.340], with no interaction [*F*_(2, 58)_ = 0.127, *p* = 0.817, $\eta_{p}^{2}$ = 0.004]. The RT for MT and LT was significantly longer than that for UT (MT vs. UT = 678.806 ms vs. 664.641 ms, *p* = 0.009; LT vs. UT = 690.820 ms vs. 664.641 ms, *p* < 0.001). In terms of ACC, there was no main effect for both Prime [*F*_(1, 29)_ = 2.801, *p* = 0.105, $\eta_{p}^{2}$ = 0.088] and Probe [*F*_(2, 58)_ = 1.010, *p* = 0.371, $\eta_{p}^{2}$ = 0.034] nor the interaction between the two factors [*F*_(2, 58)_ = 0.954, *p* = 0.391, $\eta_{p}^{2}$ = 0.032].
